# Logo therapy effect on anxiety and depression in mothers of children with cancer

**Published:** 2014-04-20

**Authors:** H Delavari, M Nasirian, K Baezegar bafrooei

**Affiliations:** 1 Departmment of clinical psychology, college of human science, Yazd science and research branch, Islamic Azad university, Yazd, Iran; 2 psychiatrist, Departmment of clinical psychology, college of human science, Yazd science and research branch, Islamic Azad university, Yazd, Iran; 3 Assistant professor, Yazd University, Yazd, Iran

**Keywords:** Logo Therapy, Depression, Anxiety, Children with Cancer

## Abstract

**Background:**

Cancer diagnosis among children can cause high stress and anxiety in parents, and they may lose their life expectancy. The present study investigated the effectiveness of Logo therapy on anxiety and depression among mothers of children with cancer.

**Materials and Methods:**

This study was conducted by a semi-pilot method using pre-test and post-test with a control experimental group. Therapy sessions were held during 9 sessions of Logo therapy training for 90 minutes. The participants of this study were selected among 30 mothers of children with cancer and using sampling method in Yazd hospitals. The participants divided randomly into two groups: experimental and control. Participants in both experimental and control group completed questionnaires on Beck Anxiety Inventory and Beck Depression Inventory before and after training.

**Results:**

The results showed that the index of depression and anxiety in control and experimental groups are 32.3, 6.63, 7.4 and 6.75, respectively. So, the level among the experimental group has been decreased after intervention of Logotherapy training and a significant difference occurred in the pre-post test stages. The results showed that Logo Therapy has a significant effect in reducing anxiety and depression among mothers of children with cancer (p<0.05).

**Conclusion:**

Regarding the efficiency of this approach to reduce anxiety and depression among mothers, this treatment is recommended to be practiced beside other cancer therapies, so they can practice the treatment process with a better mood and mentality.

## Introduction

One of the main causes of child mortality in developed and developing countries is cancer. This disease is included about 4 percent of deaths in children 5 to 15 years among Iranian population (1).Principally, mothers of children with cancer will be faced with a plagued experience in their families, once it is diagnosed, they suffer with infertility and have to live with a burden as their child’s disease will affect their families and overall quality of life declines (2).The crisis caused by the child's illness and hospitalization impact on all family members (3).Fear and anxiety are common feelings among parents that their child is in the hospital (4).

A lot of studies have shown that existence of sick children as a stressor in the family, have a significant effect on parents’ depression. Children are more in contact with their mothers than any other members in the family so mothers are exposed at the greatest risk for stress and depression (5).

Litzelman and Gangnon claimed that the stress caused by the child's condition is the reason for decrease of life quality among parents of children with cancer (6). Medicines and its side effects beside the stress and anxiety that comes into the family, also puts pressure on families. Providing nonpharmacologic treatments such as psychological interventions for child and his parents would be effective to accept the reality of this disease (7).

Therefore, supportive care with different methods of counseling is an integral part of patients’ treatment; in addition, their families should not be neglected (8).

Logotherapy is one of the most important approaches of existential psychotherapy or humanoriented that focuses on the spiritual aspect of human and the meaning of human existence. Logotherapy is based on this idea that if suffering is an integral part of life, so there is a meaning in it, and when a person’s suffering is meaningful, it will not be annoying anymore. That’s why this ideology is entitled Logotherapy (9).

Consultant helps client to access the highest possible vital activity. Client is helped not only to experience the universe and display a continuous exploring of the potential values, but also is committed to accept responsibility and undertake a specific task (10). He needs to change negative attitudes (my child will die turns to my child will recover), and he helps himself (I am the one who can deal with this problem). Finally, he must look for acquiring meaning in life and in what is causing his suffering (depression and anxiety symptoms caused by the fact that the child has cancer). Therefore, it can be expected that using Logo Therapy along with 4 techniques such as Paradoxical intention, Dereflection, Attitude Correction, and Elicitation Techniques by the therapist during the treatment process can reduce the symptoms of depression and anxiety (9).

Logotherapy among other methods is the one that provides an excellent philosophical and theoretical field for teamwork. Hence, it can be used for intervention purposes in the best situation (11). Since the Logotherapy has effect on quality of life and public health; therefore, the researcher aims to find whether Logotherapy is effective in the reduction of anxiety and depression among mothers of children with cancer.

## Materials and Methods

This study is a practical research, conducted by a semi-pilot method using pre-test and post-test with control and experimental groups. The participants of this study were 30 mothers of children with cancer, hospitalized in Shahid Sadoughi hospital in Yazd. The researcher divided 15 mothers randomly in the experimental group and 15 mothers in control group. Participants in both groups before any intervention, completed Beck Depression Inventory (BDI) and Beck Anxiety Inventory (BAI). Both groups were asked to complete questionnaire after therapy sessions again (9 treatment sessions on experimental group).

**Data collection materials:**

Beck Depression Inventory (BDI):

This 21-question multiple-choice instrument is a self-report questionnaire to measure the severity of depression in adults and adolescents with 13 years old and older. 

The questions of this questionnaire consisted of sadness, sense of failure, dissatisfaction, sense of guilt, punishment expectation, self-dislike, self-accusation, suicidal thoughts, crying, irritability, social isolation, indecision, physical change imagination, incapability, insomnia, fatigue, appetite change, weight loss, preoccupation and loss of sexual interest.

The sum of scores in this test ranges from 0 to 62, so a negative score is not included. These rates have been proposed for diagnosis of depression (it should be noted that these values are listed in two different ways).

1- Normal Average (1-15) or (1-18): People who are not depressed and stand in a normal level.

2-Mild depression (16-31) or (18-28): People who are suffering from a mild depression level.

3-Moderate depression (32-47) or (29-35): People who are in a moderately depression level. 

4-Deep depression (48-62) or (36-63): People who are suffering from severe depression.

The psychometric studies which were conducted on the second edition of this questionnaire have shown a good reliability and validity. Beck have been reported the internal consistency of the instrument, 73% to 92% (12).

The diagnostic value of this test in Iran was approved by Vahabzadeh to examine the diagnostic value of this test among depressed patients and healthy individuals. (13).also approved the validity and reliability of this test by an experimental study on the distribution of depression among college students of Tehran University. He modulated this test on the Iranian population level (14). 

Beck Anxiety Inventory (BAI):

The questionnaire consisted of 21 items and symptoms of anxiety. Participants should have chosen one of the four options that reflected his anxiety, as "never", "strong", "moderate" and "slight". Four options of each question have been scored on a range of four-part from a scale of zero to three. Each item of questionnaire describes the common anxiety symptoms (mental and physical symptoms and fear). The total score of this questionnaire ranges from zero to 63. The questionnaire is developed in a way that is not included depressive symptoms (15). Validity and reliability of Beck anxiety Inventory were assessed by Kaviani on Iranian patients and healthy population. The result showed the validity of (R=0.72), reliability (R=0.83) and internal consistency (alpha 0.92) on Iranian population (20). The validity of 0.90 was reported in a research, and the correlation coefficient of this questionnaire is obtained by physiological parameters 0.89 (16).

The following segmentation has been proposed for cut points of this questionnaire by an Iranian study (Kaviani et al) 0 to 11 asymptomatic, 12 to 18 slight, 9 to 26 moderate, 27 to 36 strong, and 37 to 63 very strong.

**Intervention method:**

Logotherapy training held for 9 consecutive sessions over two days (weekly meetings). 

The criteria were considered for the present study is as follows:

-Parents age between 20 to 45 years

-Disprove any initial psychiatric disorders in association with major depressive disorder

-No side problems such as serious marital problems

-People in Logotherapy were not taking any medication

-Absence of not more than 2 sessions during training sessions

-Non- participation in the test

The training details briefly shows in Table I (17).

**Statistical Analysis**

Statistical analyzes was performed by SPSS software. The results was considered significant when p value <0.05.

## Result

Descriptive statistics for the study, including average, and standard deviation of all the variables are presented in Table II.

Table II shows that depression and anxiety level among the experimental group has been decreased after intervention of Logotherapy training and a significant difference occurred in the pre-post test stages. However, these conditions were similar for the control group and no changes have occurred.

Levine's test results in Table III showed the homogeneity of variance in depression and anxiety condition. The analysis of covariance in table IV shows the effect of Logotherapy training in reducing the anxiety and depression level in mothers of children with cancer. Table IV shows that the effect of pre-test was statistically significant (p< 0.05) and this significance is the result of pre-test removal effect on pos-test (p<0.05).Therefore, the test result shows that Logotherapy training reduces anxiety and depression and the effectiveness range of this therapy is almost 20% on anxiety and 53% on depression.

**Table I:** Logotherapy training sessions

**1st session **	Referrals (getting familiar with other group members and the therapist) - informing the clients about the fundamental concepts of Logotherapy
**2nd session**	Statement of the problem and assessment of the quality of a child's life from the perspective of children and mothers - discussion about the most important element of Logotherapy (to live a meaningful life)
**3rd session**	Describing of anxiety and depression, understanding the factors that cause anxiety and depression, identifying mothers’ belief to cancer
**4th session**	Expression of expected anxious –Reverse psychologytechnique and its application in practice
**5th Session**	Discussion in the field of individual freedom, responsibility and linking between the vision and the reality of life
**6th session**	Perception of suffering meaning – creation of a sense through pain and suffering - expression of reflex therapy techniques
**7th session**	Expression of attitudes correction techniques - positive thinking discussion
**8th session**	Perception and understanding of death meaning - Discussion in the field of transient life - the fact of death and its meaning
**9th Session**	Summary and conclusion of discussed subjects, the understanding of the clients - Answer to questions/FAQ

**Table II:** Standard deviation and variables variance of anxiety and depression

**Groups**	**Index**	**Depression**		**Anxiety **	
		**Pre-test**	**Post-test**	**Pre-test**	**Post-test**
**Control**	Average	32.40	32.3	17.26	7.4
	Standard deviation	13.95	12.95	16.73	6.16
**Experimental**	Average	27.47	6.63	17.80	4.38
	Standard deviation	19.60	6.87	13.27	6.75

**Table III:** Levine Test in depression and anxiety condition

	**F**	**Df 1**	**Df 2 **	**Significance level**
**Depression**	1.37	1	28	0.25
**Anxiety**	1.96	1	28	0.17

**Table IV:** Covariance Test

	**Sum of Squares. Type III**	**F Degree**	**Square **	**F**	**Significance level**	**Rate**
**Depression level. Pre-test**	2560.41	1	2560.41	13	0.004	**0.85**
**Group**	503.73	1	503.73	30.23	0.005	**0.53**
**Anxiety rate.** ** Pre-test**	725.81	1	725.81	44.13	0.005	**0.62**
**Group**	114.61	1	114.61	6.97	0.01	**0.20**

## Discussion

Based on findings, Logo therapy method has been effective to reduce anxiety and depression among mothers of children with cancer. The results of the present study is compatible with the results of Fakhar (18), Faranoushet al (19), Khanjari (20-22), Mousaviet al (23), Asghary and dadkhah (24), Abolghasemi et al (25), Haghighi (26), and Southwick (27).

A family that has a child with cancer faces high anxiety and the anxiety level is greater among mothers. Khanjari et al (22). Study also showed that there is a significant relation between gender (being father or mother) and quality of parents’ life. The reason of this relation has been mentioned as anxiety and stress caused by the unpredictable nature of the disease, treatment uncertainty and other mothers’ responsibilities at home and community that has been paralleled with the present study.

In addition, it was shown in this study that mothers bear more responsibility for their own child, so despite high anxiety level they are depressed as well. Therefore, this high anxiety and depression will have effect on child's treatment procedure. Parental concerns were high so that they focused on the quality of child's life much less than the child herself/himself (intervention in this study was based on understanding of physical appearance and communication).

The parents whom their children were under chemotherapy in Philadelphia hospitals, in the USA, also reported the quality of their children’s life less than the children themselves. 

The present study also was compatible with Faranoush et al(19) (throughPeds QL generic core scale questionnaire).Due to differences in parents and children’s beliefs about cancer, Logotherapy protocol can alter this belief. The present study has shown the alternation of mothers’ belief and growth of life expectancy as a result of anxiety and depression reduction. 

Kyo and Suhhoo also reported that Logotherapy and doing exercise have had significant effect on life activities and eventuated to a meaningful life and self-esteem (28). 

In this study, it was shown that Logotherapy training enables mothers to realize the meaning of life suffering and it changes their attitudes towards life, society and bearing, so it leads them to a better understanding and acceptance of true purpose of life and their child’s disease. 

Study of Kang Kyung on the effectiveness of Logotherapy on adolescents showed that Logotherapy has been effective on decrease of pain and increase of life meaning among adolescents with cancer (11). He showed that the body weakness and inefficiency feelings of patients with cancer in comparison with other society people; cause a lack of confidence and low self-esteem. If Logotherapy and psychological interventions can give the sense of ability and control back to them, so it enables them to enter the community with a better mood, and they will have a higher chance for recovery and depression decrease.

Therapist in this study concluded that acceptance of freedom and awareness of being finite leads to anxiety inevitably, and this result was compatible with Mohammadi findings as well. Fear of losing child or other cancer-related disturbances such as costs, duration of treatment and relapse have been the outcomes of anxiety and depression in this study. Khanjari et al also reported that child’s exhausting care, financial, social and family problems have been the reasons of low quality of life for parents of children with cancer (21).

According to the reported findings, psychological counseling is recommended along with cancer treatment to improve the patients and entourage’s mental situation. Organizing medical treatment groups to support the family, and the presence of whole family can help patient’s treatment. In addition, financial support to reduce the treatment costs also can be helpful to solve some of patients’ problems.

## Conclusion

The results showed that group Logotherapy has a significant effect on reducing anxiety and depression in mothers of children with cancer. So, it is necessary to pay more attention to effective nonpharmacologic treatments forreducing anxiety and depression. Group Logotherapy also can be an effective aid to mothers.

## Conflict of interest

The authors have no conflict of interest.
